# Fatal Errors

**Published:** 1881-08

**Authors:** Ad. Lippe

**Affiliations:** Philadelphia


### FATAL ERRORS.

AD. LIPPE, M. D., PHILADELPHIA.

IT is a fatal error to teach that the totality of symptoms means the pathological state which we have before us, be it a functional or already an abnormal organic one; that to prescribe for the totality of symptoms is a superficial method; that we may be lost in a wilderness of symptoms if we fail to consider the pathological characteristic which gives us the key-note to all the other symptoms.

This fatal error has been committed by the learned editor of the *North American Journal of Homœopathy*, committed over and over again, in the May No., 1881, but most boldly asserted on page 585. Has the learned editor of that journal, who is also a public teacher in the homœopathic college at New York, never seen a work entitled "The Organon of the Healing Art," by Samuel Hahnemann, the founder of a "System of the Healing Art," by him called Homœopathy? Can a man claim to be able to teach homœopathy when he is evidently ignorant of its teachings? How does he, this learned teacher, propose to apply the fundamental law of the similars, when he, firstly, considers the pathological condition a characteristic key-note to all the other symptoms; and, secondly, tries to find among the effects of medicines on the human organism, a similar characteristic key-note—a pathological condition? The pathological condition of the sick, a hypothesis, as well as the pathological condition of the prover, also a hypothesis to be made subservient to the law of the similars. This is really and truly the newest of the late progressively increasing departures; this is the boldest attempt to annihilate all of Hahnemann's teachings, and supplant them by old and obsolete absurdities.

Hahnemann tells us in his Organon, paragraph 13, "Disease, therefore, (those forms of it not belonging to manual surgery,) considered, as it is by allopathists, as *something* separate from the human organism, and the vital principle which animates it as something hidden internally and material, how subtile soever its nature may be supposed, is a nonentity which could only be conceived by minds of material mould, and which for ages, hitherto, has given to medicine all those pernicious deviations which constitute it a mischievous art." Does our learned friend think it possible to put the pathological livery upon that which homœopathicians term the totality of the symptoms? Shall we be led back to partake of the fleshpots of Egypt? The pathological characteristics are the least important symptoms in a given case of disease; the symptoms belonging to the sick individual characteristically are of much more importance; and again, will our learned friend give us permission to illustrate?

The illustration is best taken from actual cases. Here is a young man down with typhus fever. The twenty-first day finds him extremely ill; the diagnosis is mortification of stomach and intestines; he vomits up great quantities of black masses; his bowels are frequently moved; color of the fæces black and extremely offensive; pulse very frequent and small. Here then, according to this modern teacher, are the characteristic key-notes of a pathological condition. Now, Mr. Professor, what is the remedy? There is no time to lose; it seems as if this is to be the last prescription for better, for worse. Of course, says our learned friend, in the pathological livery, sporting a scientific cane, here is a clear case for Arsenic; but Arsenic has been given, and he is rapidly growing worse. As a last resort, the true healer plunges into the wilderness of symptoms, finding that to prescribe for a pathological condition is the superficial, and therefore unsuccessful, way to prescribe homœopathically. Among that wilderness of symptoms appears a singular one, by no means necessarily present or necessarily belonging to this unmistakable pathological condition, and it is *this*, the patient while his skin is cold desires, nay demands, to be entirely uncovered; the room is cold, but he will have no cover. It is now clearly discernible why *Arsenicum*, which is very similar in its sick-making properties, and which has caused just such a pathological condition, was of no use whatsoever. The characteristic symptom of Arsenic is, *amelioration from heat;* but this patient despises, is made worse from heat. Out of that wilderness of symptoms, which totality of symptoms is such a stumbling block to the scientific physician in the physiologico-pathological livery, we see a glimpse of light arising, and we again turn to our Materia Medica, and there find that *Secale Cornutum*, which has just the opposite symptom from Arsenic, viz.: *aggravation from heat*, from being covered, has likewise the similar pathological condition, and because it corresponds with the characteristic symptoms of the patient, it is now administered in the smallest attainable dose, and the patient recovers. Where is the man with his pathological livery? And what of Hahnemann's methods? what of the wilderness of symptoms outside the pathological key-note? What does our learned friend really want? May we guess? Is he quietly paving the way so that we may be lulled into following the pathological track still further? is he paving the way for the isopathists? It really looks that way. What a glorious future there is in prospect! This patient has syphilis, therefore must he have *Syphilinum* very high. The other fellow has or had gonorrhœa, and *Medorrhin.*, extremely high, must he have; or he suffers stomachache from eating of the cucumber or of any other food, say lobster, and forthwith the fountain is set to work, and bottlewashing enables the learned healer to produce a sky-high potency of the injurious food, and hereafter, *i. e.*, after a few pellets have touched the tongue of the sufferer, not only all pains cease, but he may from that day forward partake with impunity of the food which he never dared touch before without severe suffering. The inventor or discoverer of this superior mode of healing will set aside Hahnemann, his pains-taking therapeutics, his inductive method, and, mounted on the pathological race-horse, will ride rough-shod over the master and all who follow him. Will the editor of the *Quarterly* then fire a salute? will he tell his readers that the Millennium has come in the resurrection of poor *Lux?*

